# Professional-Grade TCA-Lactic Acid Chemical Peel: Elucidating Mode of Action to Treat Photoaging and Hyperpigmentation

**DOI:** 10.3389/fmed.2021.617068

**Published:** 2021-02-12

**Authors:** Vinay Bhardwaj, Krati Sharma, Srdjan Maksimovic, Aixing Fan, Alison Adams-Woodford, Junhong Mao

**Affiliations:** ^1^Department of Global Personal Care and Skin Health R&D, Colgate-Palmolive Company, Piscataway, NJ, United States; ^2^Independent Researcher, Philadelphia, PA, United States; ^3^Physicians Care Alliance (PCA) SKIN, Scottsdale, AZ, United States

**Keywords:** chemical peeling, molecular docking, melanin, collagen, anti-pigmentation, photo-aging

## Abstract

Chemical peeling is usually performed by dermatologists, plastic surgeons, and aestheticians for the treatment of photo-aged skin, dyspigmented skin, skin prone to acne eruption, and pre-cancerous skin lesions, etc. In this research paper, we report our investigative findings to understand the mode of action of a commercial professional chemical peel to treat hyperpigmented and photoaged skin. In the *in-vitro* experiments, we found that the peel inhibits enzymes that are responsible for degradation of collagen and elastin, and the production of melanin pigment. It was surprising to observe that trichloroacetic acid (TCA), which is considered a workhorse of chemical peels for its cauterant action, could synergistically promote the inhibitory action of lactic acid. The rationale behind this synergistic effect could be the conformational change in TCA from linear structure to ring-like structure, which was elucidated through sequential docking using Rosetta software. The *in-vitro* results on collagen and elastin were corroborated by up-regulation of COL1A, COL3B, fibronectin, and elastin gene expression from 3D human skin equivalents treated with the peel. The findings were further validated through *ex-vivo* testing on human skin biopsy. The peel significantly inhibits the production of total melanin, and ameliorates photo-damage that was evident through repair of the collagen in the skin exposed to a biological effective dose of UV daily light (6 J/cm^2^). These research findings have implications for product developers and users (dermatologists, plastic surgeons, and aestheticians) in improving safety and efficacy of chemical peels/peeling.

## Introduction

Chemical peeling is a non-surgical dermatology procedure that is most often performed by a licensed skincare professional in a medical spa or a physician in their clinic. According to the year 2019 surveys, the American Society of Plastic Surgeons (1) and the American Society for Aesthetic Plastic Surgery (2) reports chemical peeling among the most popular minimally-invasive non-surgical procedures in the United States. The procedure involves use of chemicals to exfoliate “chemoexfoliation” dead or damaged cells from the skin surface to reveal new and healthy cells, in a process called cell turnover or skin rejuvenation. Although the user will not necessarily experience visible exfoliation after peeling, the active ingredients in peels are still working at a cellular level. The treatment of sun damage (photo-aging) and unevenly pigmented skin (dyspigmentation) are two primary indications of chemical peeling intervention (2, 3). Although in recent years new exfoliation techniques like laser, dermabrasion, etc. are invented, chemical peeling remains most popular owing to minimal side-effects, ease of performance and relatively lower cost (3). Trichloroacetic acid (TCA) and Alpha Hydroxy Acids (AHAs) are well-known ingredients used in the chemical peels for their caustic and exfoliating effects (4–6). In addition to the aforementioned mode of action involving exfoliation, another theory behind chemical peeling is their ability to induce controlled wound healing or injury through their acidic effect, thereby triggering the skin's defense system to stimulate key components of the extracellular matrix like collagen, elastin, and hyaluronic acid. This acidic effect could be our foe or friend based on the concentrations of the acids being used in the peel (5).

To achieve desired depth and benefits with minimum side-effects, it is recommended to use TCA at lower concentration by combining it with other peeling agents, such as Jessner's solution and/or hydroxy acids (7). For example, a superficial peel consisting of <30–35% TCA and 10–70% hydroxy acids (alpha and/or beta) could achieve depth of penetration into papillary dermis, and can treat mild photoaging, acne scarring and pigmentary disorders (3, 7–9). Although there is scarcity of literature on adversity of TCA-based chemical peels, there are possibilities of post-inflammatory hyperpigmentation and acid burn (7). Therefore, peeling should be performed by a trained professional or physician and a post-operative care should be followed to prevent complications associated with peeling agents (7). A typical post-operative care regimen includes application of gentle cleansing, aquaphor ointment, moisturization, and sunscreen application to protect and nourish exfoliating skin (9). Although there is no universal protocol, several dermatologists prefer to use topical retinoids, such as tretinoin and retinol, hydroquinone, etc., pre- and post-peel to achieve optimum outcomes and safety (7, 8, 10). Chances of complications and post-operative care are minimum with superficial peels. TCA is most commonly used in combination with AHAs, such as lactic acid and glycolic acid (7) because mechanism of action of AHAs and their indications are well-understood (11, 12). AHAs are indicated for use in many in-home and dermatologists' cosmetic and medical products for skin moisturization, wrinkle and pigment reduction, and chemical peeling (3, 12). More recently, lactic acid has emerged as safer alternate to gold-standard glycolic acid without compromising efficacy. Lactic acid is structurally similar to glycolic acid with a difference of additional methyl group, and a lower pKa and pH than glycolic acid (3). Thus, lower pH of lactic means it is required at much lower concentration than glyclolic acid to achieve equivalent efficacy (keratocoagulation), neutralization is not necessary with lactic acid, and the recovery time is quick (3). Depending on the strength of the acids in the peel, chemical peeling can be broadly categorized as at-home, professional, and physician's use only. The strength or concentration of the active ingredients in professional and physician's peels is prescription level and hence not available for retail sale or at-home use. Depending on skin conditions and sensitivity, a dermatologist will pick professional or physician's peels from one of these three depths: (1) Superficial peels that usually do not penetrate beyond the papillary dermis and usually contain active ingredients, such as TCA at <30–35% (3, 7–9) and often combined with other peeling agents like alpha or beta hydroxy acids, and retinoids, such as tretinoin and retinol, etc. at low levels (10, 11), (2) medium-depth peels that penetrate to the papillary dermis, usually containing trichloroacetic acid (TCA) at low to medium concentration, and (3) deep peels that penetrate to mid-reticular dermis and usually contains phenol or TCA at medium to high concentration (3). TCA remains the workhorse of chemical peels for the dermatology standard of care because of its well-understood cauterant mode of action to facilitate penetration to deeper layers of the skin and its proven track record of safety (4). Although TCA and TCA blended AHA peels remain dermatologists' standard of care for the treatment of a wide range of skin conditions from pigmentary dyschromias to photo-aging, not much is known about TCA activity other than its cauterant action, and its interaction with AHA. The objective of this research is to investigate mode of action of a superficial TCA-AHA chemical peel to treat pigmentation and photo-aging.

In this research paper, we investigated (1) efficacy of a commercial professional-grade TCA-lactic acid chemical peel to treat pigmented and photo-aged skin, (2) synergistic inhibition activities between TCA and lactic acid, and (3) mechanism of inhibition through molecular docking.

## Methods

Commercial peel was obtained from PCA Skin®. Human skin biopsy was obtained from abdominal plastic surgery after informed consent of the donor.

### *In-vitro*: Enzyme Inhibition Assays

The test product (chemical peel) and its main active ingredients (TCA and lactic acid) were tested at very low concentration and after neutralizing the pH. Peels were diluted 10× before their serial dilution testing. For synergy testing, neat working stocks of TCA, lactic acid, and their mixture was prepared at 1:2, i.e., and 0.312% TCA and 0.625% lactic acid. The working stocks were diluted serially to investigate the synergy at three doses. The peel and the active ingredients were tested using kits from Sigma Aldrich for their activities to inhibit tyrosinase (enzyme responsible for production of melanin pigment, catalog#MAK257), collagenase (enzyme responsible for degradation of collagen, catalog#MAK293), and elastase (enzyme responsible for degradation of elastin, catalog#MAK213), following manufacturer's protocol. Briefly, positive control and test samples were added to the enzyme solutions, and allowed to incubate for at least 15 min at room temperature. Enzyme control (negative control) was prepared by adding buffer in place of test samples. Following incubation, substrate solution was added and the 96 well plate was read immediately using a plate reader (Spectramax M5e from Molecular Devices, USA) at 510 nm for tyrosinase, 345 nm for collagenase, and 400Ex/505Em for neutrophil elastase. Readings were taken every 5 min for at least 30 min to plot a kinetic curve and obtain slope of the curve. The percent relative inhibition with respect to enzyme control was calculated using this equation: [(Slope of Enzyme Control – Slope of Test Samples)/slope of Enzyme Control] × 100. A dose-dependent study (serial dilution) and positive controls were run to validate the results. The pH of all the samples including test agents, positive and negative controls was measured at the end of the final reading (pH was 7.2 ± 0.2) to rule out any effect of acidity on the enzymes activity. We used the positive controls agents that were well-studied: kojic acid for tyrosinase (13), 1,10 phenanthroline for collagenase and N-(Methoxysuccinyl)-Ala-Ala-Pro-Val-Chloromethyl Ketone (SPCK) for elastase (14).

### Synergy Investigation and Computation

A mixture of TCA and lactic acid was tested at ratio 1:2 (as it exists in the test peel product), for above-mentioned enzyme inhibition activities. The synergy and dose reduction index (DRI) was investigated following constant ratio serial dilution method and CompuSyn software developed by Chou (15, 16).

### *In-silico* Molecular Docking

To elucidate the mechanism of tyrosinase inhibition by lactic and trichloroacetic acids, Rosetta software was used for molecular docking, and the binding energy was reported as Rosetta Energy Unit (REU). Chimera was used for energy minimization, clash removal, and structure visualization. Active Site Prediction Server from IIT, Delhi was used to compute binding pockets/cavities in the protein. Before application of the docking model on lactic acid and TCA, the robustness of the model was first tested on kojic acid, the positive control, and a benchmark compound in molecular docking for tyrosinase. Sequential docking of TCA and lactic acid was performed to gain insights on their synergistic interactions.

### *In-vitro*: 3D Human Skin Equivalents and qPCR

EpiDermFT 3D full thickness human skin equivalents (MatTek Corp, USA) were cultured in EFT400 culture media supplemented with growth factors, hormones, and lipid precursors (MatTek Corp, USA). This human skin model is bioengineered using epidermal keratinocytes and dermal fibroblasts from a single normal human donor to form a multilayered model for human epidermis and dermis. The model is characterized for metabolic and mitotic activities, and other *in-vivo*-like morphological and growth characteristics required to study anti-aging, wound healing, skin irritation, and many other properties of cosmetic and medical products (17–19). The peel was diluted to 10% solution before testing; this dose was found to be non-toxic to tissue as investigated by Alamar Blue assay. The skin equivalents were treated with buffer (control) or 10% peel solution for 1 min. RNA was isolated from the skin using RNeasy Plus Mini Kit (Qiagen Inc., USA) following manufacturer's protocol. Briefly, skin samples were lysed using lysis buffer supplied in the kit. The lysate was passed through gDNA eliminator spin column to remove DNA contaminant, followed by passing through RNeasy Minielute spin column to separate RNA that was eventually eluted in water for the next step of cDNA synthesis. cDNA was synthesized using Maxima First Strand cDNA Synthesis Kit with dsDNase (Thermo Scientific, USA) following the manufacturer's protocol. Briefly, it was a two-step procedure. First, RNA samples were incubated with dsDNase (supplied in the kit) to remove any genomic DNA. Second, enzyme mix (Maxima Reverse Transcriptase) and Reaction Mix [dNTPs, oligo (dt)18 and random hexamer primers] were added to the purified RNA from step 1. The mixture is centrifuged and incubated for 10 min at 25°C, followed by 15 min incubation at 50°C, and finally the reaction was terminated by heating the mixture at 85°C for 5 min. The cDNA was stored at −80°C unless used for Real-time qPCR (RT-qPCR). Rt-qPCR was performed using Quant Studio 7 Flex Real-Time PCR System (Applied Biosystems, USA) to quantify differential expression (ΔCt) of genetic markers for inflammation (Interleukins 1a, 6, and 8), hydration/barrier (Filagrin and Claudin1), regeneration (Connective tissue growth factor and insulin-like growth factor 1), and extracellular matrix (Collagen 1A, Collagen 3A, Elastin, and Fibronectin 1).

### *Ex-vivo*: Tissue Culture and Product Treatment

Human skin biopsies were cut into 8 × 3 mm (diameter × thickness) skin samples. Skin samples were weighed to select samples with approximately similar weight. Skin samples were cultured on top of the perforated stainless-steel rings that were in contact with modified William's E culture media. Each skin sample was covered with a 6 mm diameter membrane, and the commercial peel solution (liquid) was applied on top of the membrane to achieve uniform product application on the skin. Product was applied every day, and the culture media was changed every 3rd day until the 6th day when skin samples were harvested for viability and histochemical measurements.

### Skin Viability

Skin samples were tested for viability using 3-(4,5-dimethylthiazol-2-yl)-2,5-diphenyl tetrazolium bromide (MTT) assay. The liquid peel solution was tested in serial dilution: 1:1 (100% stock product), 1:2, 1:4, and 1:8 to identify non-toxic dose of the peel for next step, that is, modulation of melanin and collagen by peel.

### Photo-Damaged Collagen *Ex-vivo* Skin Model

To assess the efficacy of the peel in treating photo-damaged skin, UV-damaged *ex-vivo* skin model was prepared following individual typology angle (ITA)-based method to predict biological effective dose of UV (BED-UV) (20). Briefly, BIO-SUN from Vilber Lourmat was used to expose skin biopsies (donor aged 62 years and intermediate skin type with ITA 37°) with 6 J/cm^2^ UV. To mimic real-world conditions UVA (5.76 J/cm^2^) and UVB (0.24 J/cm^2^) were used in 96:4% (21), which corresponds to sun burn but without any DNA damage (20). This 6 J/cm^2^ BED-UV was found to induce only the collagen damage (but not melanin pigmentation), and hence only the photo-damaged collagen skin model was used for the study.

### Histochemical Imaging and Semi-quantification

For each treatment group (untreated, positive control, and test product “peel” after 2× dilution), skin biopsies (*N* = 6) were treated every day and then harvested on day 6 for histochemical processing: sectioning followed by staining and imaging. Two random sections from each biopsy were imaged and processed to obtain statistical information from *N* = 12 images or data points for each treatment group. For melanin imaging, sections were stained with Fontana Masson (melanin granules in black/brown/dark gray color), and for collagen sections were stained with Picrosirius Red (collagen fibers in purple-red color). Proprietary algorithm and process was used to quantify melanin and collagen content in sectioned biopsies. Briefly, images were de-convoluted to separate signals from background, for example, collagen in red/purple from non-collagen background in blue/green color. The raw RGB colored images were converted into 8-bit L*a*b color space using Image J software from National Institute of Health. The transformed L* values were normalized on the ratio between selected area and the area of the slide to produce pigmentation and collagen scores.

### Positive Controls

Kojic acid for anti-pigmentation activity (*in-silico* molecular docking, *in-vitro* anti-tyrosinase assay, and *ex-vivo* melanin inhibition), 1,10 phenanthroline for *in-vitro* anti-collagenase assay, retinol for *ex-vivo* collagen synthesis, and SPCK for *in-vitro* anti-elastase assay.

### Statistics

For statistics at least two independent experiments were done for all experiments. Student *t*-test was done for significance testing on *in-vitro* data. One-way ANOVA with permutation test followed by Tukey's or Dunnett's and *t*-test followed by permutation was done on *ex-vivo* data to evaluate significant differences between different groups. *P*-value <0.05 was accepted for significance difference.

## Results

### *In-vitro* Enzyme Inhibition Activities

**Figure 1:**
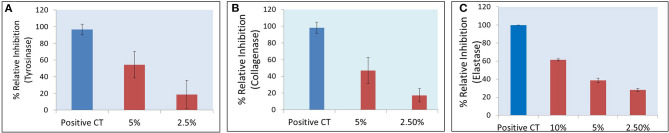
A significant (*P* < 0.05) dose-dependent decrease in tyrosinase **(A)**, collagenase **(B)**, and elastase **(C)** activities after peel treatment is observed, peel diluted at 10, 5, and 2.5% v/v. Positive controls are kojic acid for tyrosinase (2 mM), 1,10 phenanthroline for collagenase (2 mM), and N-(Methoxysuccinyl)-Ala-Ala-Pro-Val-chloromethyl ketone (SPCK) for elastase (0.6 mM).

**Figure 2:**
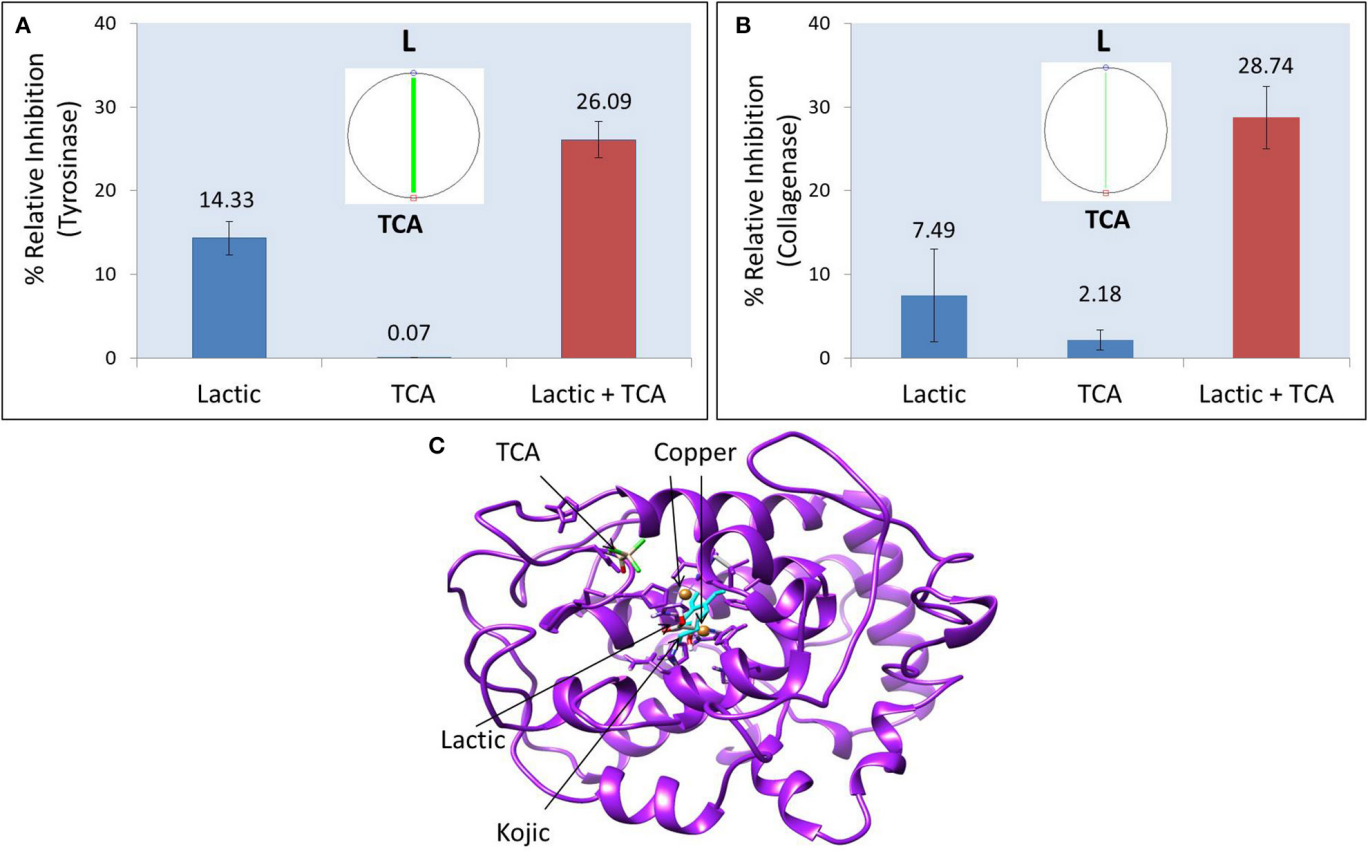
Synergistic effect of TCA:lactic at ratio 1:2 for inhibitory effect on tyrosinase **(A)** and collagenase **(B)**. Insets show polgonograms computed by CompuSyn showing synergy. Crystal structure of tyrosinase showing docked structures of lactic, kojic and TCA **(C)**. The concentration of TCA and lactic acid in tyrosinase synergy testing is 0.156 and 0.312%, respectively and for collagenase synergy testing the concentration is 0.625% TCA and 1.25% lactic acid.

The commercial peel was found to significantly inhibit tyrosinase (Figure 1A), collagenase (Figure 1B), and elastase (Figure 1C) in dose-dependent manner. Compared to collagenase and tyrosinase, the elastase assay had relatively higher reproducibility (evident from tight error bars), as the latter was fluorescence assay, while the other two were based on absorbance. TCA and lactic acid (the active ingredients in the peel) were further tested as neat solutions to investigate any possible synergy. TCA:lactic at ratio 1:2 were found to exhibit strong synergy for anti-tyrosinase (Figure 2A) and anti-collagenase activities (Figure 2B). This is the optimum ratio to achieve maximum synergy as we observed a decrease in synergy when deviated from this ratio. The results using CompuSyn further confirmed that it is not an additive effect but a true synergy between TCA:lactic at 1:2 for anti-tyrosinase (Figure 2A, inset) and anti-collagenase (Figure 2B, inset). The intensity of the green line in CompuSyn polygonograms (insets) indicates strength of the synergy. No synergy was observed for anti-elastase activity. Figure 2A and the computational results from CompuSyn (Supplementary Table 1) insinuate that TCA has little to no tyrosinase inhibition activity, however, TCA is critical for synergy and it can reduce dose of the TCA and lactic acids (denoted by DRI “dose reduction index” in Supplementary Table 1) by several folds to achieve the same level of efficacy (denoted by Fa “fractional activity” in the Supplementary Table 1).

### *In-silico* Molecular Docking to Elucidate Mechanism of Tyrosinase Inhibition

Tyrosinase is the key enzyme responsible for pigmentation. The structure of tyrosinase enzyme and its mechanisms of inhibition by benchmarks ingredients, such as kojic acid and hydroquinone for hyper- and dyspigmented skin are well-characterized (22).

Figure 2C shows the crystal structure of tyrosinase. The catalytic site of tyrosinase (active site responsible for production of melanin from tyrosinase) is characterized by the presence of two copper atoms that are essential for the catalytic activity of the enzyme. The superimposition of native kojic acid with retrieved kojic acid (Supplementary Figure 1A), and the similarity in their binding energy and binding residues (Table 1) with previous reports validate the robustness of our model (13, 22). This validated model was used to dock TCA, lactic acid, and their sequential docking. Kojic acid shows highest affinity for tyrosinase inhibition (−6.686 REU), followed by lactic acid (−5.496 REU), and TCA (−2.149 REU) (Table 1). Compared to kojic and lactic acid which bind at the catalytic site (Figure 2C), and share the same hydrophobic cavity (Supplementary Figure 1B), TCA showed maximum affinity away from the catalytic site (Figure 2C). When TCA and lactic acid were docked sequentially, surprisingly, a change in structure from linear to ring-like structure was observed in TCA (Supplementary Figures 1C,D). This change to ring-like structure could be the rationale behind the synergistic effect between TCA and lactic acid, and needs further investigation.

**Table 1:** Binding or inhibition properties of Kojic acid (positive control), lactic acid, and trichloroacetic acid (TCA) to tyrosinase enzyme, docked using Rosetta software.

**Ligand or ingredient**	**Type of inhibitor**	**Binding energy (REU)**	**Binding residues**	**H bonds**
Kojic acid	Catalytic	−6.686	**ARG 205,209, ASN 201,205, HIS 208,204**,200, 56, 60, MET 211, 215, ALA217,221	HIS 56, MET 211
Lactic acid	Catalytic	−5.496	**ARG 205,209, ASN 201,205, HIS 208,204**, GLY 212,216, VAl213,217	ARG 205, ASN 201
Trichloroacetic acid	Allosteric	−2.149	GLY 42, 46, MET 57,61, ASN 53,57, VAL 214, 218	0

*The common binding residues of kojic acid and lactic acid are highlighted bold*.

### *In-vitro* 3D Human Skin Model

The commercial peel solution was found to exhibit no significant effect on metabolic inhibition of bioengineered 3D human skin model. There was no significant change (Fold change <2) in expression of IL-6, IL-8, IL-1a, FLG, CLDN1 between peel treated vs. buffer-treated skin. No change in these genetic markers of inflammation and skin barrier indicates the peel is mild with no adverse biological response. However, there was at least a 2-fold increase in COL1A, FN1, COL3A, ELN, CTGF, and IGF1 (data not shown). An increase in these ECM components (collagen, elastin, and fibronectin) and growth factors (CTGF and IGF1) supports the peel's mode of action behind anti-aging and skin regeneration.

### *Ex-vivo* Human Biopsy

**Figure 3:**
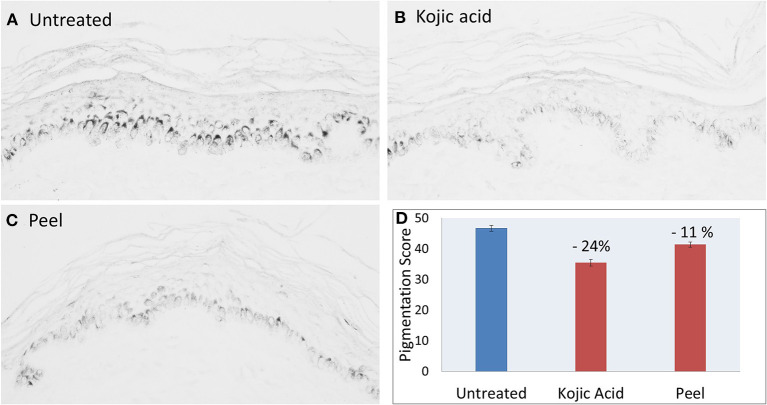
Histochemical images **(A–C)** and quantification **(D)** showing melanin granules in gray and melanin content of skin sections before (untreated) and after treatment with kojic acid (positive control, 0.1%) or peel (after 2× dilution). *P*-value <0.05 peel vs. untreated.

The commercial peel product was tested at 50% (2× dilution of commercial product) as it was found to induce no inhibition of metabolic activity of the human biopsies at this concentration (Supplementary Figure 2). Figure 3 shows images of skin sections after Fontana Masson staining for melanin imaging and semi-quantification of the melanin content (black/dark gray granules) in the skin: Untreated vs. treated with product or positive control (kojic acid). The integrity of skin structure and abundance of melanin-containing cells in the basal layer of epidermis is clearly visible. There is a clear decrease in black/dark gray intensity of melanin granules after treatment of the skin with peel (50% solution) and positive control (0.1% kojic acid). Similar observations were made in all six different skin samples/pieces tested for each condition. Supplementary Table 2 shows melanin scores/values of five biopsies, two sections from each biopsy (excluding two outliers from 6th biopsy) for every treatment condition. There was significant change, −11 and −24% decrease in pigmentation score (melanin content) of the skin after treatment with peel and kojic acid, respectively (Figure 3D).

**Figure 4:**
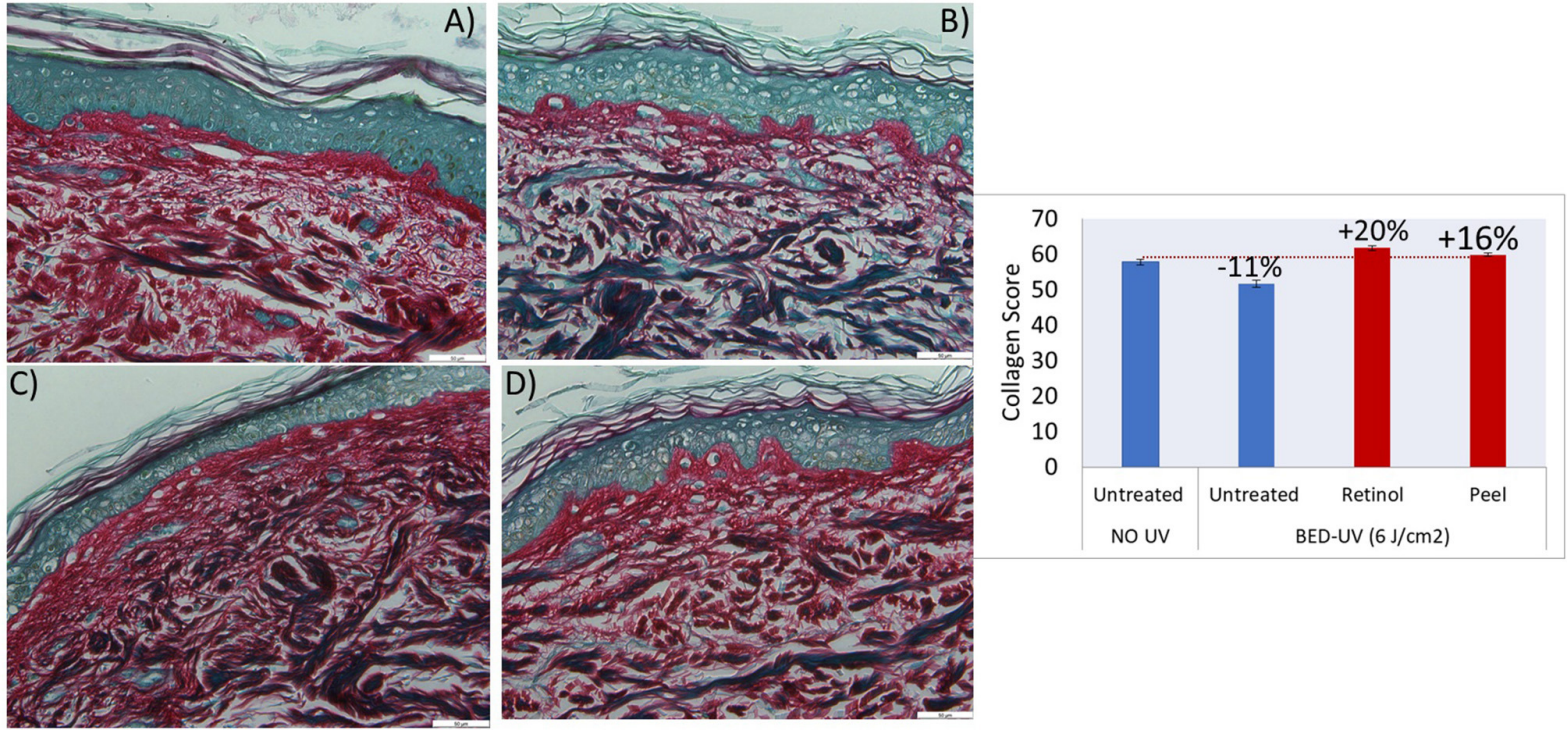
Histochemical images of skin biopsies showing collagen in purple-red color, before treatment “no UV and no product” **(A)**, after exposure to BED-UV **(B)**, treatment of BED-UV exposed skin with retinol as positive control **(C)** and peel **(D)**. The graph shows significant change (recovery) of BED-UV damaged collagen (−11%) after treatment with retinol (positive control, 0.05%) or peel (after 2× dilution). *P*-value <0.05 peel vs. untreated BED-UV.

Figure 4 shows images of skin sections after Picosirius Red staining of the skin biopsies, untreated (A) exposed to a BED of UV daylight (B) and UV-damaged skin treated with positive control 0.05% Retinol (C) or Peel (D). Purple-red colored collagen fibers and non-purple anatomical structures, such as hair follicles and glands, etc. are observed. Photo-damage in response to UV is indicated through breakdown of collagen which is evident through its non-native organization (Figure 4), and decrease in content (Figure 4). The subsequent repair of this UV-damaged collagen by peel and positive control retinol is indicated through re-organization of damaged collagen to native stat (Figures 4C,D), and an increase in collagen content from −11 to +20% and +16% for retinol and peel, respectively. Supplementary Table 3 shows the statistical results from collagen scores/values of 6 biopsies (2 sections from each biopsy) for every treatment condition.

## Conclusions and Discussion

Chemical peeling is among the most common non-surgical aesthetic procedures that costs an average of ~$644 physician/surgeon's fee with total expenditure of ~$900 million (1). TCA and AHA are the workhorses of chemical peels used as standard of care for the treatment of a wide range of skin conditions, such as pigmentary dyschromias, photo-aging, melasma, etc. (3). TCA is well-known for its caustic effect (4), while AHAs are known for exfoliation (6). However, not much is known about TCA activity other than its caustic action. Further, TCA interaction with AHA is unknown, despite the inherent importance that several chemical peels in the market contain TCA and AHA. In our research, we investigated a commercial TCA-blended lactic acid peel to understand its mechanism of action behind treatment of photo-aged and pigmented skin. We used state-of-the-art *in-vitro, in-silico*, and *ex-vivo* methods to get a clear understanding on the mode of action of the peel and its primary active ingredients TCA and lactic acid.

We found that the peel strongly inhibits tyrosinase, collagenase, and elastase, which are enzymes responsible for production of melanin pigment, and degradation of collagen and elastin, respectively (Figure 1). Surprisingly, TCA and lactic acid, which are primary active ingredients in the peels show strong anti-tyrosinase and anti-collagenase synergy at the ratio used in the peel, that is, TCA:lactic at 1:2 (Figures 2A,B). These surprising findings motivated us to use computational tools to elucidate the mechanism of inhibition and simulate the impact of this synergy on products safety and efficacy. This synergistic effect could reduce the dose of lactic acid by several folds if used with TCA at 1:2 (TCA:lactic) to achieve the same efficacy. For example (Supplementary Table 1), to achieve 90% anti-tyrosinase efficacy, it requires 2.4% lactic acid when used alone, however, it will be 4.24-fold less lactic acid (0.56%) to achieve the same 90% efficacy if at least 0.25% TCA is added to the 0.56% lactic solution. The reduction in dose due to lactic acid and TCA synergy is helpful to minimize side-effects of chemical peeling, as they are highly acidic and have caustic effect (23, 24). This simulated dose reduction index (DRI) by CompuSyn has implications in medicine to guide clinicians in selecting safe dosage for their clinical trial (15). The rationale behind the synergy between TCA and lactic acids could be the change in structure from linear to ring-like structure that we observed in sequential docking of the two ingredients with tyrosinase (Supplementary Figures 1C,D). Currently, scientists as well as industries are moving away from the traditional one- drug-one target-one disease approach in drug discovery to a more powerful synergistic approach (25). Examples of some synergistic mixtures using active ingredients including those used in chemical peels for anti-tyrosinase or anti-pigmentation activities are kojic acid and hydroquinone (26), resorcinol, resveratrol, glabridin (25), resorcinol and bisabolol (27), etc.

Further, the peel did not induce significant changes in expression of genetic biomarkers for inflammation (IL 1a, 6, 8) and skin barrier (FLG, CLDN1). Rather, it increased genetic expression of ECM components and growth factors that supports the peel's mode of action behind anti-aging and skin regeneration.

UVA is associated with skin photo-aging, while UVB is associated with sunburns (28). Therefore, to simulate real-world UV conditions for photo-aging *ex-vivo* model, human skin biopsy samples were exposed to the 6 J/cm^2^ BED of UV daylight comprising of 96% UVA and 4% UVB (21), that induced sun burn/erythema but not DNA damage (20). The degradation of collagen when exposed to BED of UV, followed by the repair of this UV-damaged collagen by peel and the positive control Retinol (Figure 4) confirms the benefit of using peel. Our results are in agreement with the previous report using UVA and UVB for photo-aging study, though conditions were unreal as UVB: UVA was 4:1 (29).

In summary, we found dual mode of action behind the action of a TCA-blended lactic acid peel. First, the peel inhibits enzymes responsible for degradation of ECM components (collagen and elastin), thereby protecting the native ECM present in our skin. Second, the peel induces expression of genes responsible for synthesis of ECM components and growth factors to stimulate skin regeneration but without any side-effects on integrity of skin barrier and skin inflammation. The peel was able to repair UV-damaged collagen and inhibits melanin pigmentation in skin biopsies. We also discovered synergistic action of TCA and lactic acids active ingredients present in the peel, which could be due to their conformational changes from linear to ring-like structures. This synergistic action has implications in chemical peels (and medicine overall) to develop safe and efficacious treatment by reducing the dose of the active ingredients.

## Data Availability Statement

The original contributions presented in the study are included in the article/Supplementary Material, further inquiries can be directed to the corresponding author/s.

## Author Contributions

VB did planning and required collaborations for the study. AF supervised the study. VB, KS, and SM performed experiments. JM and AA-W revised the manuscript. All authors reviewed and approved the final draft of the manuscript.

## Conflict of Interest

VB, SM, AF, and JM was employed by the company Colgate Palmolive. AA-W was employed by the company PCA Skin (brand of Colgate-Palmolive). The remaining author declares that the research was conducted in the absence of any commercial or financial relationships that could be construed as a potential conflict of interest.

## Supplementary Material

Supplementary Figure 1 Crystal structure of Tyrosinase showing catalytic site (indicated by presence of two copper atoms in brown), and the binding of native (cyan) and retrieved (elemental color) structures of kojic acid to the catalytic site **(A)**. Kojic (cyan) and lactic acid bind in the same hydrophobic cavity, while TCA binds into a separate cavity **(B)**. Lactic (green) and TCA (elemental) when docked independently are linear structures **(C)**, but when docked sequentially a conformational change to the ring-like new structure appears **(D)**.

Supplementary Figure 2 MTT assay showing effect of peel concentration on metabolic activity of the skin biopsies.

Supplementary Table 1 Dose reduction index (DRI) of lactic (L) and TCA simulated by CompuSyn. Fa, fractional activity (efficacy).

Supplementary Table 2 Mean melanin scores of treatment conditions after proprietary image processing (10 images for each treatment condition) to compare melanin inhibition efficacy of untreated vs. positive control and peel.

Supplementary Table 3 Mean collagen scores of treatment conditions calculated after proprietary image processing (12 images for each treatment condition) to compare collagen repair efficacy of positive control and peel vs. untreated UVDL (after UV daily light damage).
