# Supplementary figures and images for: Professional-Grade TCA-Lactic Acid Chemical Peel: Elucidating Mode of Action to Treat Photoaging and Hyperpigmentation
Source: Front Med (Lausanne). 2021 Feb 12;8:617068. doi: 10.3389/fmed.2021.617068 (PMC7928281; doi:10.3389/fmed.2021.617068)

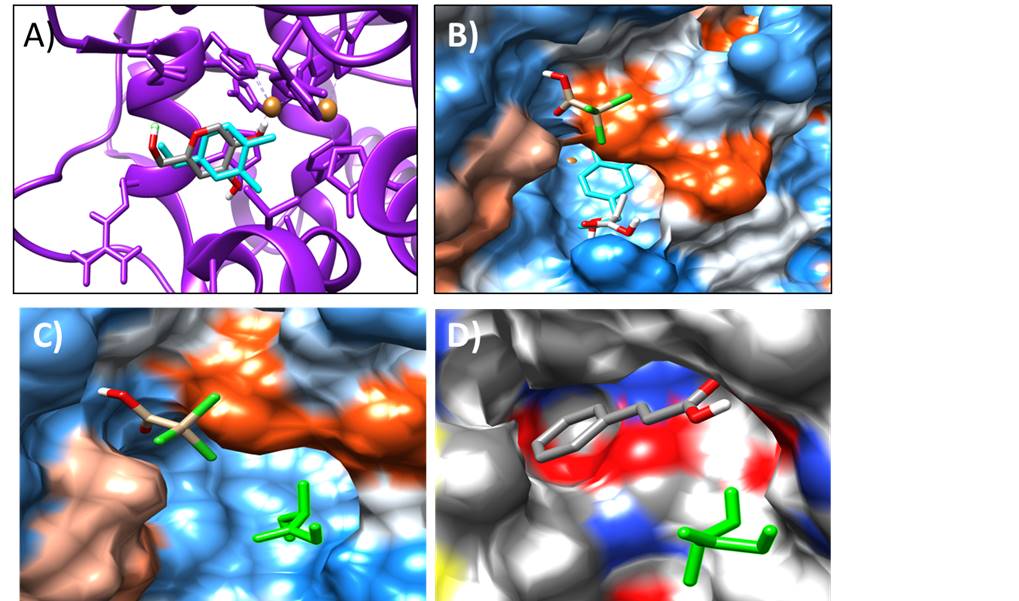

Supplement: Supplementary Figure 1 — Crystal structure of Tyrosinase showing catalytic site (indicated by presence of two copper atoms in brown), and the binding of native (cyan) and retrieved (elemental color) structures of kojic acid to the catalytic site (A). Kojic (cyan) and lactic acid bind in the same hydrophobic cavity, while TCA binds into a separate cavity (B). Lactic (green) and TCA (elemental) when docked independently are linear structures (C), but when docked sequentially a conformational change to the ring-like new structure appears (D). [file Image_1.JPEG]

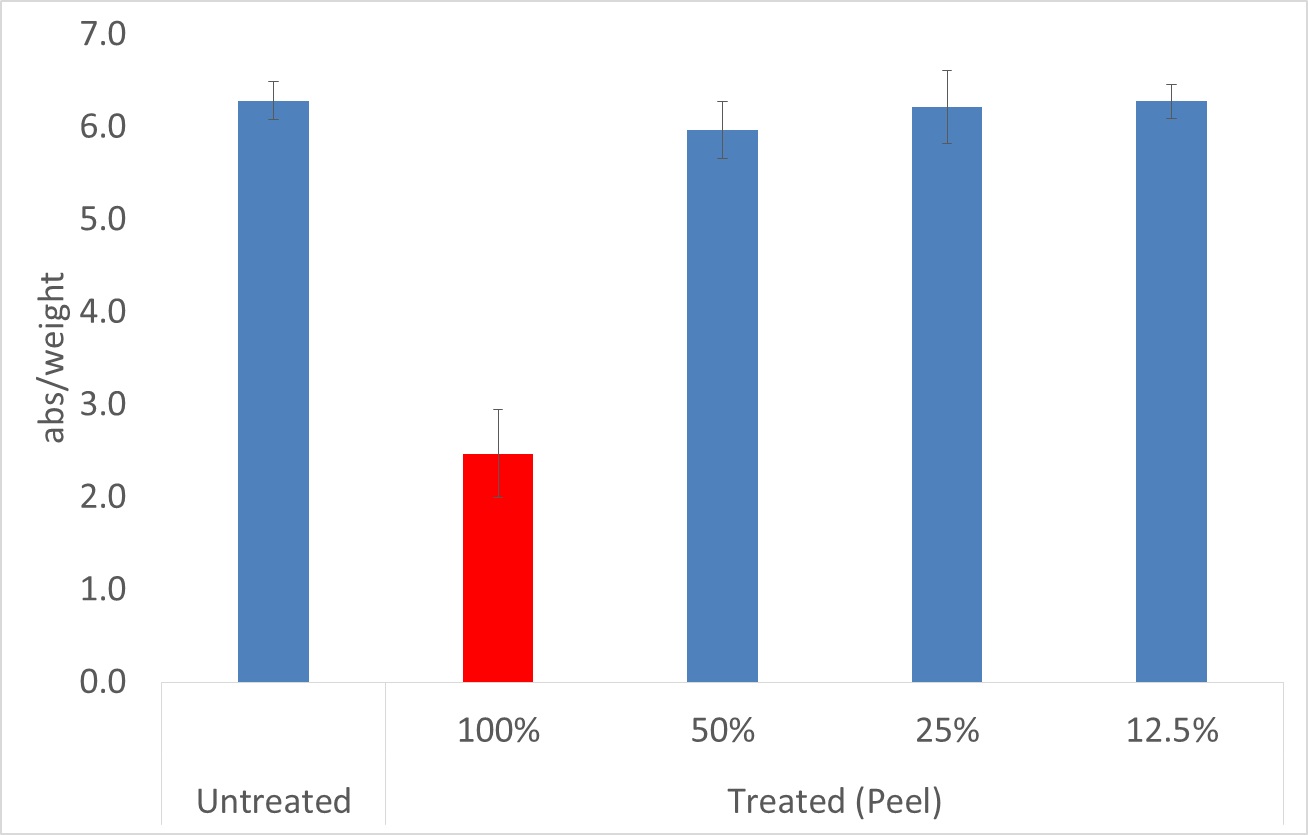

Supplement: Supplementary Figure 2 — MTT assay showing effect of peel concentration on metabolic activity of the skin biopsies. [file Image_2.JPEG]

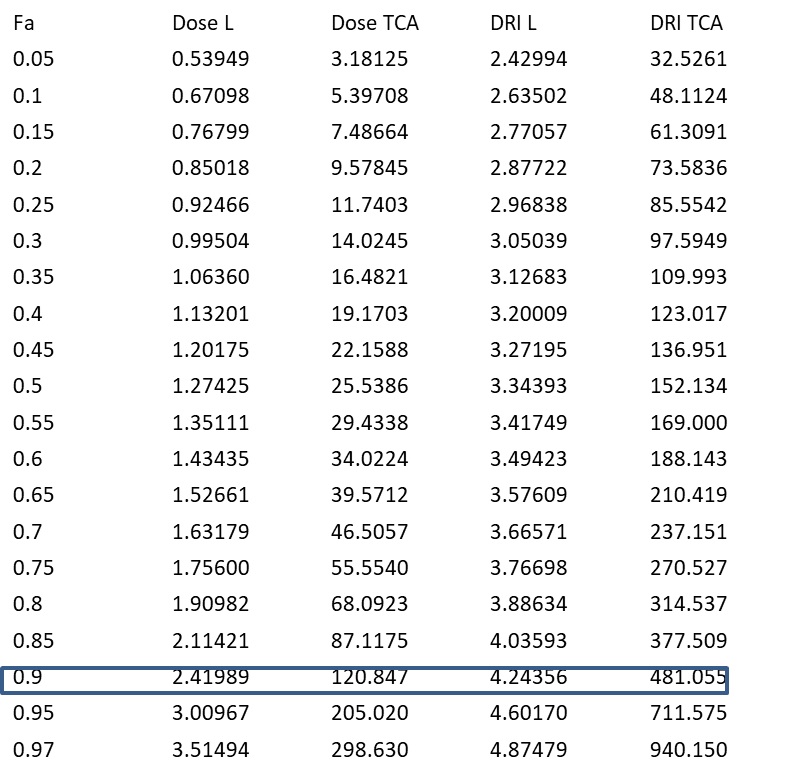

Supplement: Supplementary file 6 [file Image_3.JPEG]
