# Supplementary material for: Professional-Grade TCA-Lactic Acid Chemical Peel: Elucidating Mode of Action to Treat Photoaging and Hyperpigmentation
Source: Front Med (Lausanne). 2021 Feb 12;8:617068. doi: 10.3389/fmed.2021.617068 (PMC7928281; doi:10.3389/fmed.2021.617068)
Supplement: Supplementary Table 1 — Dose reduction index (DRI) of lactic (L) and TCA simulated by CompuSyn. Fa, fractional activity (efficacy). [file Table_1.pdf]

Supp. Table 1: Dose reduction index (DRI) of lactic (L) and TCA simulated by CompuSyn. Fa = fractional activity (efficacy).

Fa	Dose L	Dose TCA	DRI L	DRI TCA
0.05	0.53949	3.18125	2.42994	32.5261
0.1	0.67098	5.39708	2.63502	48.1124
0.15	0.76799	7.48664	2.77057	61.3091
0.2	0.85018	9.57845	2.87722	73.5836
0.25	0.92466	11.7403	2.96838	85.5542
0.3	0.99504	14.0245	3.05039	97.5949
0.35	1.06360	16.4821	3.12683	109.993
0.4	1.13201	19.1703	3.20009	123.017
0.45	1.20175	22.1588	3.27195	136.951
0.5	1.27425	25.5386	3.34393	152.134
0.55	1.35111	29.4338	3.41749	169.000
0.6	1.43435	34.0224	3.49423	188.143
0.65	1.52661	39.5712	3.57609	210.419
0.7	1.63179	46.5057	3.66571	237.151
0.75	1.75600	55.5540	3.76698	270.527
0.8	1.90982	68.0923	3.88634	314.537
0.85	2.11421	87.1175	4.03593	377.509
0.9	2.41989	120.847	4.24356	481.055
0.95	3.00967	205.020	4.60170	711.575
0.97	3.51494	298.630	4.87479	940.150
