# Supplementary material for: Professional-Grade TCA-Lactic Acid Chemical Peel: Elucidating Mode of Action to Treat Photoaging and Hyperpigmentation
Source: Front Med (Lausanne). 2021 Feb 12;8:617068. doi: 10.3389/fmed.2021.617068 (PMC7928281; doi:10.3389/fmed.2021.617068)
Supplement: Supplementary Table 2 — Mean melanin scores of treatment conditions after proprietary image processing (10 images for each treatment condition) to compare melanin inhibition efficacy of untreated vs. positive control and peel. [file Table_2.docx]

Supp. Table 2: Mean melanin scores of treatment conditions after proprietary image processing (10 images for each treatment condition) to compare melanin inhibition efficacy of untreated Vs positive control and peel.

|  | **Untreated** | **Kojic Acid (0.1%)** | **Peel (50% solution)** |
| --- | --- | --- | --- |
| **1** | 40.76 | 29.77 | 36.42 |
| **2** | 42.45 | 31.57 | 37.66 |
| **3** | 44.50 | 33.56 | 40.70 |
| **4** | 46.76 | 33.62 | 40.82 |
| **5** | 47.81 | 34.10 | 40.88 |
| **6** | 48.66 | 35.99 | 41.14 |
| **7** | 48.70 | 36.87 | 43.00 |
| **8** | 49.12 | 38.97 | 43.30 |
| **9** | 49.12 | 39.13 | 44.44 |
| **10** | 49.22 | 40.52 | 45.17 |
| **Mean** | **46.71** | **35.41** | **41.35** |
| **St.Dev** | 3.08 | 3.49 | 2.78 |
| **SEM** | 0.97 | 1.11 | 0.88 |
| **Delta% vs Untreated** |  | -24% | -11% |
