# Supplementary material for: Professional-Grade TCA-Lactic Acid Chemical Peel: Elucidating Mode of Action to Treat Photoaging and Hyperpigmentation
Source: Front Med (Lausanne). 2021 Feb 12;8:617068. doi: 10.3389/fmed.2021.617068 (PMC7928281; doi:10.3389/fmed.2021.617068)
Supplement: Supplementary Table 3 — Mean collagen scores of treatment conditions calculated after proprietary image processing (12 images for each treatment condition) to compare collagen repair efficacy of positive control and peel vs. untreated UVDL (after UV daily light damage). [file Table_3.docx]

Supp. Table 3: Mean collagen scores of treatment conditions calculated after proprietary image processing (12 images for each treatment condition) to compare collagen repair efficacy of positive control and peel Vs untreated UVDL (after UV daily light damage)

|  | **NO UVDL** | **0.8 BED UVDL** | | | |
| --- | --- | --- | --- | --- | --- |
|  | **Untreated** | **Untreated** | **Retinol (0.05%)** | **Peel (50% solution)** |  |
| **1** | 62.70 | 54.31 | 61.10 | 57.70 |  |
| **2** | 54.78 | 53.62 | 60.56 | 59.18 |  |
| **3** | 56.49 | 45.05 | 64.43 | 57.86 |  |
| **4** | 60.41 | 54.19 | 62.60 | 60.02 |  |
| **5** | 58.31 | 50.42 | 62.30 | 59.69 |  |
| **6** | 54.48 | 52.08 | 62.93 | 58.20 |  |
| **7** | 57.81 | 53.13 | 60.19 | 61.13 |  |
| **8** | 58.85 | 52.67 | 64.96 | 59.94 |  |
| **9** | 56.26 | 44.78 | 60.56 | 62.42 |  |
| **10** | 62.17 | 51.46 | 63.60 | 62.15 |  |
| **11** | 53.73 | 52.26 | 58.90 | 60.10 |  |
| **12** | 58.01 | 55.59 | 58.56 | Outlier |  |
| **Mean** | **57.83** | **51.63** | **61.72** | **59.85** |  |
| **St.Dev** | 2.89 | 3.42 | 2.08 | 1.60 |  |
| **SEM** | 0.84 | 0.99 | 0.60 | 0.48 |  |
| **Delta% vs Untreated NO UVDL** |  | -11% | 7% | 3% |  |
| **Delta% vs Untreated UVDL** |  |  | 20% | 16% |  |
